# Predictive models and early postoperative recurrence evaluation for hepatocellular carcinoma based on gadoxetic acid-enhanced MR imaging

**DOI:** 10.1186/s13244-022-01359-5

**Published:** 2023-01-08

**Authors:** Qian Li, Yi Wei, Tong Zhang, Feng Che, Shan Yao, Cong Wang, Dandan Shi, Hehan Tang, Bin Song

**Affiliations:** 1 Department of Radiology, Sichuan University, West China Hospital, No. 37, GUOXUE Alley, Chengdu, 610041 Sichuan Province People’s Republic of China; 2 Department of Radiology, Henan Provincial People’s Hospital, Zhengzhou, Henan Province People’s Republic of China; 3 Department of Radiology, Sanya People’s Hospital, Sanya, 572000 People’s Republic of China

**Keywords:** Hepatocellular carcinoma, Early postoperative recurrence, Machine learning, Gadoxetic acid-enhanced MR imaging

## Abstract

**Background:**

The prognosis of hepatocellular carcinoma (HCC) is still poor largely due to the high incidence of recurrence. We aimed to develop and validate predictive models of early postoperative recurrence for HCC using clinical and gadoxetic acid-enhanced magnetic resonance (MR) imaging-based findings.

**Methods:**

In this retrospective case-control study, 209 HCC patients, who underwent gadoxetic acid-enhanced MR imaging before curative-intent resection, were enrolled. Boruta algorithm and backward stepwise selection with Akaike information criterion (AIC) were used for variables selection Random forest, Gradient-Boosted decision tree and logistic regression model analysis were used for model development. The area under the receiver operating characteristic curve (AUC), calibration plots, and decision curve analysis were used to evaluate model’s performance.

**Results:**

One random forest model with Boruta algorithm (RF-Boruta) was developed consisting of preoperative serum ALT and AFP levels and six MRI findings, while preoperative serum AST and AFP levels and four MRI findings were included in one logistic regression model with backward stepwise selection method (Logistic-AIC).The two predictive models demonstrated good discrimination performance in both the training set (RF-Boruta: AUC, 0.820; Logistic-AIC: AUC, 0.853), internal validation set (RF-Boruta: AUC, 0.857, Logistic-AIC: AUC, 0.812) and external validation set(RF-Boruta: AUC, 0.805, Logistic-AIC: AUC, 0.789). Besides, in both the internal validation and external validation sets, the RF-Boruta model outperformed Barcelona Clinic Liver Cancer (BCLC) stage (*p* < 0.05).

**Conclusions:**

The RF-Boruta and Logistic-AIC models with good prediction performance for early postoperative recurrence may lead to optimal and comprehensive treatment approaches, and further improve the prognosis of HCC after resection.

**Supplementary Information:**

The online version contains supplementary material available at 10.1186/s13244-022-01359-5.

## Key points

1. HCC patients suffering early postoperative recurrence have reduced survival than those without early postoperative recurrence.
2. The preoperative models for early postoperative recurrence prediction achieved good performance.
3. These models may help the clinician to go on treatment optimization and improve HCC prognosis.

**Supplementary Information:**

The online version contains supplementary material available at 10.1186/s13244-022-01359-5.

## Background

Hepatocellular carcinoma (HCC), the most common primary liver cancer, has become the sixth most common cancer and the third leading cause of cancer-related death worldwide in 2020 [1]. Although liver resection offers the best curative approach for hepatocellular carcinoma (HCC), its prognosis remains dismal due to the high incidence of postoperative recurrence [2]. Early postoperative recurrence (i.e., recurrence within one year after surgery) is a common type of recurrence [3]. Hence, it is of vital importance to identify preoperative relevant risk factors of early postoperative recurrence and to make risk stratification and treatment optimization to improve overall prognosis. For instance, identifying HCC patients with a high risk of early postoperative recurrence is important to construct individualized surveillance strategies during the postoperative period, and even alternative treatment strategies are recommended for these patients, including neoadjuvant therapy or other nonsurgical treatment modalities. While HCC patients with a low risk of early postoperative recurrence are more suitable for liver resection and regular surveillance strategies following resection.

Various staging systems and prognostic models have been used to predict the prognostic outcomes of HCC after resection. However, of these provided methods, there are still some inherent limitations. Firstly, the various clinical staging systems are too complex in clinical practice and each system has its own unique indications [4]. Secondly, different staging and prognostic systems are developed from different populations and the risk factors are quite different. Most importantly, most clinical models are developed based on postoperative pathological factors, and it is difficult for these models to go on the prognostic prediction before surgery. Thus, non-invasive preoperative models which can provide comprehensive assessment and prediction of early postoperative recurrence for HCC are urgently needed.

Gadoxetic acid disodium is a liver-specific contrast agent that has been reported to achieve comparable diagnostic performance with extracellular contrast agent while providing additional hepatobiliary phase (HBP). Evidence [5, 6] has proven that some imaging features based on gadoxetic acid-enhanced MR imaging, such as satellite nodule and peritumoral hypointensity, can be used as non-invasive risk factors for the evaluation of aggressive biological behaviors; thus, they might also further predict the prognosis of HCC. Indeed, one radiomics nomogram [7] based on gadoxetic acid-enhanced MR imaging has achieved good discriminative performance (AUC = 0.844, 95% CI, 0.769–0.919). However, texture features are sensitive to variations in acquisition parameters, patient immobilization and organ movements [8]. There is no universal segmentation algorithm with high stability and reproducibility for many lesions with a non-smooth margin [8]. The above two challenges have limited the implementation of radiomics in routine clinical practice. To our knowledge, no study has investigated the utility of the clinical and gadoxetic acid-enhanced magnetic resonance (MR) imaging-based features to develop and validate the early postoperative recurrence predictive models for HCC.

In this study, we aimed to develop and validate the preoperative prediction models for early postoperative recurrence of HCC using clinical and gadoxetic acid-enhanced magnetic resonance (MR) imaging-based findings.

## Methods

### Patient cohort

**Fig. 1:**
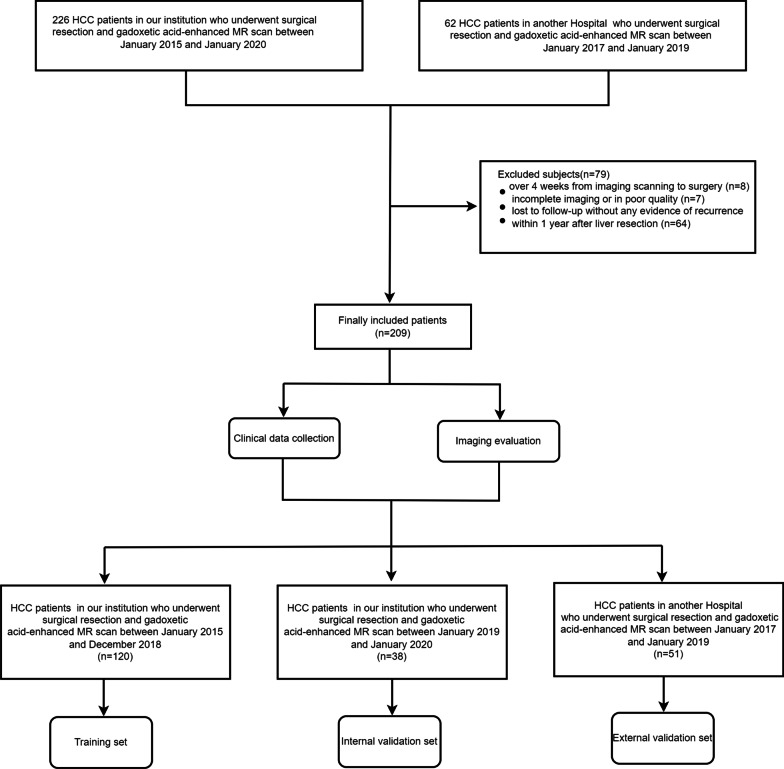
The Flowchart for the study population

This retrospective study was in accordance with the ethical guidelines of the Declaration of Helsinki, and ethical approval prior to the study commencing was granted after the review by the Institutional Review Board at West China Hospital,

Sichuan University. The requirement for written informed consent was waived from all patients. Patients (*n* = 226) in our institution from January 2015 to January 2020 and patients (*n* = 62) in another hospital between January 2017 and January 2019 who were histopathologically confirmed to have HCC, underwent gadoxetic acid-enhanced MR scan and the first-time curative-intent resection were consecutively enrolled. The inclusion criteria were as follows: (1) patients were aged ≥ 18 years; (2) the time interval between preoperative gadoxetic acid-enhanced MR imaging and surgery was less than 4 weeks; (3) primary liver lesions without any preoperative treatment (primary liver lesions referred to the initial one with no evidence of primary tumor elsewhere on imaging rather than the recurrent liver tumor.); (4) the final diagnosis was histopathologically confirmed as HCC (the histopathological proof was based on surgical resection rather than biopsy). Additionally, patients who were excluded because of (1) incomplete or poor-quality MRI images (*n* = 7); or (2) died within 1 year after liver resection and without any evidence of recurrence within 1 year after liver resection (*n* = 7) or lost to follow-up without any evidence of recurrence within 1 year after liver resection (*n* = 57), or (3) the time interval was more than 4 weeks (*n* = 8). Finally, 209 patients were enrolled. Of those, 120 consecutive HCC patients in West China Hospital (median age 52 years, range 26–75 years), including 98 men and 22 women between August 2015 and December 2018 constituted a training set; in addition, 38 consecutive patients in West China Hospital (median age 54 years, range 31–74 years), including 32 men and 6 women between January 2019 and January 2020, comprised the test set (Fig. 1). For external validation, 51 HCC patients from another hospital (Henan Provincial People Hospital) who met the above inclusion criteria were included as the external validation group between January 2017 and January 2019. Preoperative clinical and demographic data, including sex, age, Child-Pugh classification, and laboratory serum examination were also recorded. Major hepatectomy was defined as the resection of 3 or more Couinaud segments and minor hepatectomy was defined as the resection of less than 3 Couinaud segments [9]. Tumor margin was divided into the following three types: R0 resection with a wide margin (defined as removal of all microscopic and macroscopic tumors with a microscopically clear margin of surgical specimen and the resection margin was more than 1 cm), R0 resection with a narrow margin (defined as removal of all microscopic and macroscopic tumors with a microscopically clear margin of surgical specimen and the resection margin was in the range of 1 mm to 1 cm), and R1 resection with a disease-free margin which was less than 1 mm [10, 11]. The recurrence pattern was divided into three types: intrahepatic recurrence, extrahepatic recurrence and intra-plus extrahepatic recurrence [10].

### Imaging acquisition

All the patients were performed on a 3.0 T MR scanner (Magnetom Skyra, Siemens Healthineers; MR 750, GE Healthcare), and an 18-channel torsor array coil was used for all multiparametric liver MR imaging. In this study, all the patients were prepared to fast for 6–8 h before the MR examination. For the plain MR imaging, a breath-hold T2-weighted fat-suppressed fast spin-echo sequence, in- and out of T1-weighted gradient-recalled-echo sequence, and diffusion-weighted sequence (b value: 0, 50, 500, 800, 1000, and 1200 s/mm^2^) were conducted. After the plain scanning, the dynamic contrast-enhanced MR imaging with arterial phase (20–35 s), portal venous phase (60–70 s), transitional phase (3–5 min) and hepatobiliary phase (20 min) performed with the hepatobiliary specific contrast agents (Primovist®; Bayer Schering Pharma AG, Berlin, Germany). The contrast agent was administered intravenously at a dose of 0.025 mmol/kg with a rate of 1–2 mL/s and followed immediately by a 20 mL of 0.9% saline flush. The timing for arterial phase imaging was determined using an automated contrast material bolus tracking technique (7 s after the arrival of the contrast bolus in the celiac trunk) or multiple arterial phase imaging technique (acquired with 18 s long breath hold with a 20 s delay after the contrast injection and further reconstructed with a temporal resolution of 3 s). Detailed MRI parameters of each acquisition sequence are listed in Additional file 1.

### Imaging analysis

Three board-certified abdominal radiologists (readers with 8, 10 and 12 years of experience in abdominal imaging, respectively), who were blinded to the clinical, pathologic and follow-up results, independently interpreted the MR images. Prior to the review process, three reviewers discussed and selected the imaging features using 10 representative cases, and imaging features of LI-RADS v2018 [12] were used as the reference. All the three radiologists were suggested to read the MR images in a clinical manner so that the coronal and sagittal reconstructions can be used. For independent review, the consensus information was identified as follows: for binary imaging features, the value on which two or more reviewers agreed was considered as the consensus data. For multi-categorical imaging features, the value on which two or more reviewers agreed was considered as the consensus data, if the three readers got three different values, they discussed and consulted a more experienced radiologist (with 30 years of experience in abdominal imaging) until a final consensus was generated. For the size of the largest tumor, the mean value measured by the three readers was considered as the consensus data The evaluated imaging features were listed and defined in Additional file 1.

### Follow-up surveillance

Routine postoperative tumor surveillance methods consisted of imaging methods (CT or MRI) and serum tumor biomarkers including alpha-fetoprotein (AFP) and carcinoembryonic antigen (CEA). The surveillance was performed one month after surgery, and then every 3–6 months thereafter. Recurrence was defined as suspicious or positive findings on surveillance imaging or histologically confirmed disease (Due to that all the patients went on curative-intent resection with disease-free margin, the recurrent lesions were from recurrence rather than the residual tumor.). The early postoperative recurrence was defined as the incidence of recurrence within 1 year after liver resection, and the overall survival (OS) was also collected. And the last follow-up date was January 30th, 2021 in our institution, and December 1st, 2021 in another Hospital. For patients who were alive at the latest follow-up, the data of the surviving patients were censored.

### Variables selection and models development

In order to avoid over-fitting, we adopted Boruta algorithm [13] to go on variables selection for machine learning approach. This method would iteratively remove less important features than random probes until the importance is assigned for all predictors or the algorithm has reached the previously set number of random forest runs, which is 500 in our simulations. *p* value was set as 0.01. *Random forest* (RF) and *Gradient-Boosted Decision Tree* (GBDT) methods were used for the model’s development. In this study, a grid search strategy with tenfold cross-validation with five times repetition was used to identify the best combination of hyperparameters and the area under the receiver operating characteristic curve (AUC) was used to evaluate the model’s performance [14].

### Statistical analysis

Categorical variables were summarized as frequencies and proportions, while continuous variables were expressed as means and standard deviations or medians and interquartile ranges (IQRs). Analysis of variance (*ANOVA*) or *Kruskal–Wallis test* was used for the comparisons of continuous variables, *while Chi-squared test or Fisher’s exact test* was used for the comparisons of categorical variables. Bonferroni corrections were then performed for the multiple pairwise comparisons. *Fleiss Kappa* statistics or *Kendall’s W* test were used to assess the inter-observer agreements. A kappa value of 0 indicates no agreement; 0.01–0.20, slight agreement; 0.21–0.40, fair agreement; 0.41–0.60, moderate agreement; 0.61–0.80, good agreement; 0.81–1.0, perfect agreement. Survival curves of OS were drawn by using the *Kaplan–Meier* method, and the difference in OS between groups was compared using the *log-rank* test.

For these models using variables set from *Boruta algorithm*, tenfold cross-validation was also used for internal validation, and the model with the best discrimination performance was chosen for training on the entire training dataset. Meanwhile, based on the variables significant (*p* < 0.05) on bivariate analysis, a multivariable logistical regression model with the backward stepwise selection method was also trained with 10-cross validation with five times repetition to select the final variables set, and the final logistic model was trained on the total training dataset. Then, the prediction power of the two models were compared with the reference model (the BCLC stage). The discriminative performances of the prediction models were quantified by *the area under the receiver operator characteristic (ROC) curves (AUC)*. Differences in the ROC curves were compared by using the Delong test. *Calibration* curves were generated to assess the calibration of the prediction models. The probabilities of net benefits were quantified by *decision curve analysis (DCA)* to evaluate the clinical application value of the prediction models. The statistical analyses were implemented using R statistical software and SPSS software (version 22.0, IBM). A two-sided *p* values < 0.05 were considered significant.

## Results

### Patient characteristics

**Fig. 2:**
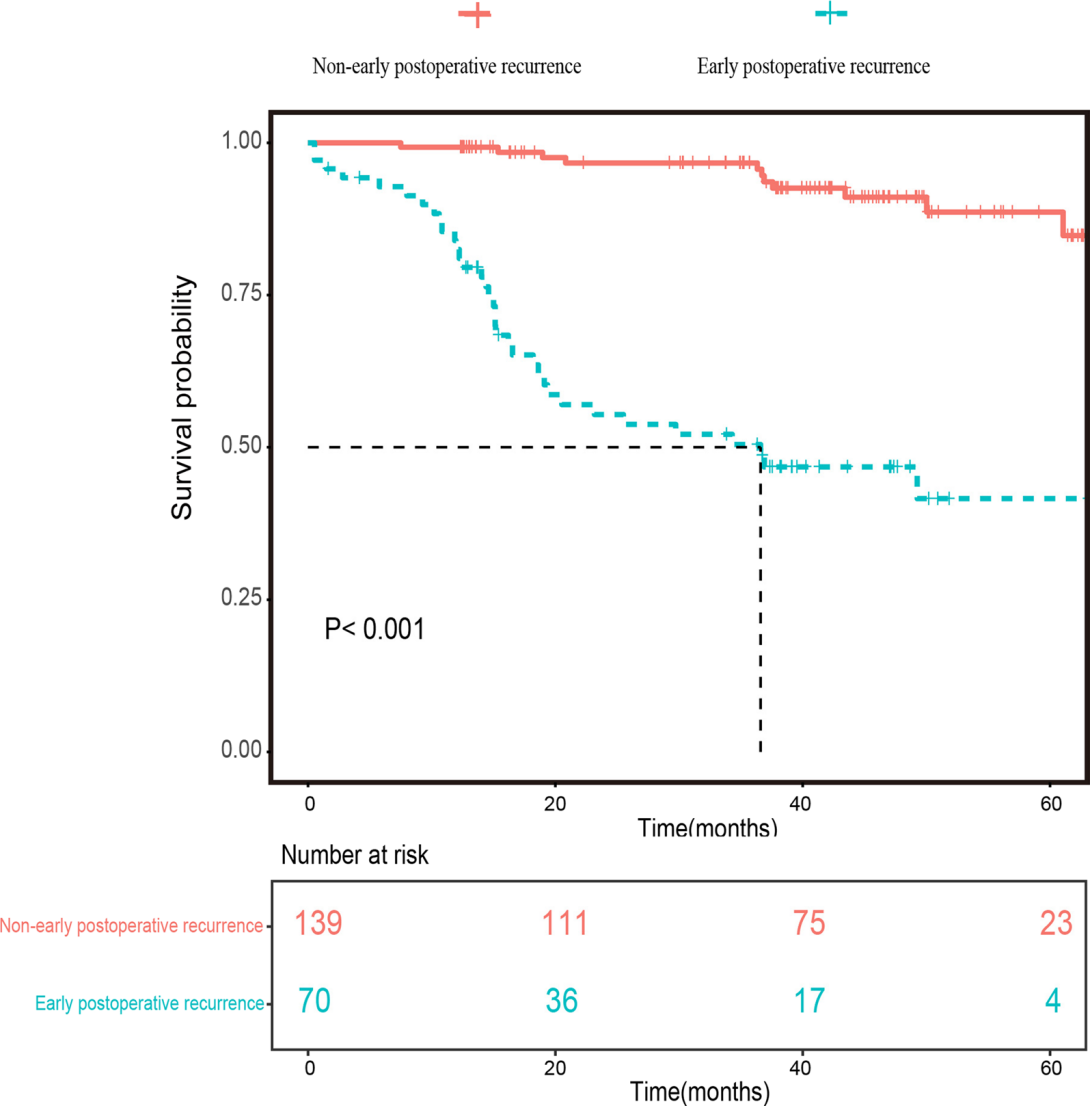
Kaplan–Meier curves of patients’ overall survival according to patients with and without Early postoperative recurrence

In the total cohort, except for the 70 patients undergoing early postoperative recurrence, 35 patients suffered recurrence beyond one year after surgery over a median follow-up period of 38.27 months (range 1.50–65.80 months). Patients without early postoperative recurrence showed longer survival (mean OS: 62.06 months, IQR, 59.91–64.20 months; 2-year OS rate: 67.62%) than those with early postoperative recurrence (mean OS: 38.38 months, IQR, 32.11–44.65 months; 3-year OS rate: 42.86%) (*p* < 0.001, Fig. 2). In the training cohort, there were 84 patients (mean age: 52.56 ± 11.08 years) without early postoperative recurrence and 36 patients with early postoperative recurrence (mean age: 48.44 ± 11.19 years). The internal validation cohort included 28 patients without early postoperative recurrence (mean age: 57.03 ± 9.43 years) and 10 patients with early postoperative recurrence (mean age: 46.98 ± 9.86 years). The external validation cohort consisted of 27 patients without early postoperative recurrence (mean age: 57.70 ± 9.48 years) and 24 patients with early postoperative recurrence (mean age: 54.17 ± 11.21 years). The rates of early postoperative recurrence of the three cohorts were similar (training cohort: 30% vs. internal validation cohort: 27.78% vs. external validation cohort: 47.06%, *p* = 0.056). According to *Kaplan–Meier analysis*, whether in the training set, internal validation set or external validation set, patients without early postoperative recurrence showed a significantly higher overall survival rate than those with early postoperative recurrence (all *p* < 0.001, Additional file 1: Fig. E1). The clinicopathological characteristics and imaging findings of the training, internal validation and external validation sets are summarized in Tables 1 and 2,* respectively*.

**Table 1:** Clinicopathological characteristics of the training, internal validation and external validation sets

Variables	Training set (*n* = 120)	Internal validation set (*n* = 38)	External validation set (*n* = 51)	*p* value
*Clinical characteristics*				
Early postoperative recurrence (within 1 year after liver resection)	36 (30.00)	10 (26.32)	24 (47.06)	0.056
Age (years)	51.22 ± 11.23	53.74 ± 10.77	56.04 ± 10.48^#^	0.034*
ALT (IU/l)	50.67 ± 64.70	44.79 ± 25.26	35.48 ± 20.92	0.199
AST (IU/l)	45.17 ± 33.37	41.55 ± 23.52	38.79 ± 24.68	0.383
PT(s)	12.34 ± 1.07^&^	11.79 ± 1.03^	13.47 ± 1.20^#^	< 0.001*
PLT (× 10^9/l)	152.30 ± 73.36	149.32 ± 68.67	138.94 ± 71.07	0.310
Gender (male/female)	68/16	32/6	44/7	0.713
AFP (> 400 ng/mL)	39 (32.5)	12 (31.58)	11 (21.57)	
CEA (> 5 ng/mL)	11 (9.17)	4 (10.53)	2 (3.92)	
HBsAg (Positive)	89 (74.17)	35 (92.11)	41 (80.39)	
ALBI grade				0.036*
A	92 (76.67)	30 (78.95)	30 (58.82)	
B	28 (23.33)	8 (21.05)	21 (41.18)	
Child-pugh				0.619
A	118 (98.33)	37 (97.37)	49 (96.08)	
B	2 (1.67)	1 (2.63)	2 (3.92)	
BCLC stage				0.001*
0	6 (5) ^&^	6 (15.79) ^	2 (3.92) ^#^	
A	56 (46.67)	14 (36.84)	44 (86.27)	
B	30 (25)	6 (15.79)	5 (9.81)	
C	28 (23.33)	12 (31.57)	0 (0.00)	
Type of surgery				0.084
Major hepatectomy(≥ 3 segments)	7 (5.83)	7 (18.42)	5 (21.57)	
Minor hepatectomy(< 3 segments)	113 (94.17)	31 (81.58)	46 (78.43)	
*Pathological characteristics*				
Tumor differentiation				0.383
High	5 (5.95)	2 (5.25)	5 (9.80)	
Low-mediate	115 (94.05)	36 (94.74)	46 (90.20)	
Resection margin				0.490
R0-Width margin(disease-free margin > 1 cm)	115 (95.83)	38 (100)	50 (98.04)	
R0-Narrow margin(disease-free margin 1 mm-1 cm)	4 (3.33)	0 (0.00)	1 (1.96)	
R1(disease-free margin < 1 mm)	1 (0.84)	0 (0.00)	0 (0.00)	
Pattern of Recurrence				0.592
Intrahepatic	21 (58.33)	8 (66.67)	17 (70.83)	
Extrahepatic	1 (2.78)	0 (0.00)	1 (4.17)	
Both	14 (38.89)	2 (33.33)	6 (25.00)	

Data are represented in mean ± SD or frequency (%). And Data were evaluated by Analysis of variance (ANOVA) or Kruskal–Wallis test for continuous variables and the Chi-square test or Fisher’s exact test for categorical variables

*ALT* Alanine aminotransferase, *AST* Aspartate aminotransferase, *PT* Prothrombin time, *PLT* Platelet count, *AFP* Alpha-fetoprotein, *CEA* Carcinoma embryonic antigen, *HbsAg* Hepatitis B surface antigen, *ALBI* Albumin-bilirubin grade, *BCLC* Barcelona clinic liver cancer

^*^referred to *p* < 0.05; ^&^referred to *p* < 0.016 (Training set vs Internal validation set); ^#^referred to *p* < 0.016 (Training set vs. External validation set); ^referred to *p* < 0.016 (Internal validation set vs. External validation set)

**Table 2:** Imaging findings of the training, internal validation and external validation sets

Variables	Training set (*n* = 120)	Internal validation set (*n* = 38)	External validation set (*n* = 51)	*p* value
Cirrhosis	49 (40.83)	12 (31.58) ^	34 (66.67) ^#^	0.003*
Multifocality	21 (17.5)	7 (18.42)	20 (39.22) ^#^	0.009*
Satellite lesions	44 (36.67)	12 (31.58)	26 (50.98)	0.121
Presence of non-hypervascular HBP hypointense nodules	26 (21.67)	11 (28.95)	15 (29.41)	0.458
Tumor in vein	49 (40.83)	12 (31.58)	26 (50.98)	0.179
APHE				0.879
No	1 (0.83)	0 (0.00)	0 (0.00)	
Non-rim	114 (95.00)	36 (94.74)	48 (94.12)	
Rim	5 (4.17)	2 (5.26)	3 (5.88)	
Corona enhancement	52 (43.33)	14 (36.84)	22 (43.14)	0.768
Washout				0.127
No	8 (6.67)	5 (13.16)	4 (7.84)	
Non-rim	112 (93.33)	33 (86.84)	45 (88.24)	
Rim	0 (0.00)	0 (0.00)	2 (3.92)	
“Capsule” appearance				0.734
No “capsule”	19 (15.83)	7 (18.42)	6 (11.76)	
Enhancing capsule	76 (63.33)	22 (57.89)	30 (58.82)	
Non-enhancing capsule	25 (20.83)	9 (23.68)	15 (29.41)	
Incomplete capsule	83 (69.17)	25 (65.79)	40 (78.43)	0.358
Blood products in mass	53 (44.17)	16 (42.11)	22 (43.14)	0.973
Nodule in nodule	52 (43.33)	11 (28.95)	14 (27.45)	0.077
Mosaic architecture	67 (55.83)	19 (50)	25 (49.02)	0.655
Non-smooth Tumor margin	6 (5)	1 (2.63)	2 (3.92)	0.949
Eggel’s growth classification				
Single nodular type	55 (45.83)	20 (52.63)	26 (50.98)	
Single nodule type with extra-nodular growth	49 (40.83)	10 (26.32)	20 (39.22)	
Contiguous multinodular type	16 (13.33)	8 (21.05)	5 (9.80)	
Restricted Diffusion	120 (100.00)	38 (100.00)	51 (100.00)	1.000
Mild-moderate T2 hyperintensity	120 (100)	36 (95.74)	51 (100.00)	0.578
TP hypointensity	119 (99.17)	36 (94.74)	49 (96.08)	0.213
HBP intensity			0.540	
Hypointensity	117 (97.5)	37 (97.37)	48 (94.11)	
Isointensity	1 (0.83)	1 (2.63)	2 (3.92)	
Hyperintensity	2 (1.67)	0 (0.00)	1 (1.96)	
Targetoid TP or HBP appearance	2 (1.67)	1 (2.63)	1 (1.96)	0.935
Peritumoral hypointensity on HBP	67 (55.83)	15 (39.47)^	39 (76.47) ^#^	0.002*
Size of the largest tumor(cm)	5.83 ± 3.12	5.38 ± 3.46	4.73 ± 3.52^#^	0.010*

Data are represented in mean ± SD or frequency (%). And Data were evaluated by Analysis of variance (ANOVA) or Kruskal–Wallis test for continuous variables and the Chi-square test or Fisher’s exact test for categorical variables. ^*^referred to *p* < 0.05; ^&^referred to *p* < 0.016 (Training set vs. Internal validation set); ^#^referred to *p* < 0.016 (Training set vs. External validation set); ^referred to *p* < 0.016 (Internal validation set vs. External validation set). *HBP* Hepatobiliary phase, *APHE* Arterial phase hyperenhancement, *T2WI* T2-weighted imaging, *TP* Transitional phase

### Variables selection

Good agreement was obtained for the evaluation of imaging features (*Fleiss Kappa: 0.654, Range:* 0.406–0.983) and the tumor size (Kendall’s value = 0.995) among the three reviewers (Additional file 1: Fig. E2). In the training cohort, 8 independent risk factors were selected for early postoperative recurrence model development including 2 clinical factors (ALT and AFP) and 6 imaging features (corona enhancement, peritumoral hypointense on HBP, single nodule type with extra-nodular growth, size of the largest tumor, satellite lesions, and tumor in vein) using Boruta algorithm (Additional file 1: Fig. E3). The best combination of hyperparameters of machine learning models and models selection were shown in Additional file 1. With a backward stepwise selection method using the lowest Akaike’s information criterion, 6 predictors were finally enrolled including satellite lesions (OR, 2.551; 95% CI, 0.929–7.005), corona enhancement (OR, 2.399; 95% CI, 0.794–7.246), single nodule type with extra-nodular growth (OR, 2.628; 95% CI, 1.046–7.199), peritumoral hypointense on HBP (OR, 4.646; 95% CI, 1.303–16.569), AFP (> 400 ng/mL) (OR, 3.559; 95% CI, 1.294–9.788) and AST (OR, 1.013; 95% CI, 1.000–1.026) (Table 3).

**Table 3:** Univariate and multivariate analysis results for early postoperative recurrence

Variables	Univariate analysis		Multivariable analysis	
	OR (95% CI)	*p* value	OR (95% CI)	*p* value
Cirrhosis	1.207 (0.538, 2.705)	0.648		
Multifocality	2.800 (1.254, 6.250)	0.012*		
Satellite lesions	4.405 (1.895, 10.239)	0.001*	2.551 (0.929, 7.005)	0.069
Presence of non-hypervascular HBP hypointense nodules	0.556 (0.242, 1.323)	0.189		
Tumor in vein	5.920 (2.332,15.031)	< 0.001*		
APHE				
No	–			
Non-rim	0.272 (0.043, 1.701)	0.272		
Rim	Reference			
Corona enhancement	5.800 (2.445, 13.756)	< 0.001*	2.399 (0.794, 7.246)	0.121
Washout				
No	Reference			
No-rim	1.308 (0.251, 6.811)	0.750		
Rim	-			
“Capsule” appearance		0.715		
No “capsule”	Reference			
Enhancing capsule	0.655 (0.227, 1.888)	0.433		
Non-enhancing capsule	0.807 (0.230, 2.830)	0.737		
Incomplete capsule	5.176 (1.676, 15.991)	0.004*		
Blood products in mass	2.275 (1.027, 5.041)	0.043*		
Nodule in nodule	1.727 (0.786, 3.796)	0.174		
Mosaic architecture	2.727 (1.171, 6.351)	0.020		
Non-smooth Tumor margin	2.302 (0.901, 5.882)	0.082		
Eggel’s growth classification		0.001*		
Single nodular type	Reference		Reference	
Single nodule type with extra-nodular growth	4.729 (1.781, 12.555)	0.002*	2.628 (1.046, 7.199)	0.044*
Contiguous multinodular type	8.816 (2.484, 31.289)	0.001*	–	
Restricted Diffusion	–			
Mild-moderate T2 hyperintensity	–			
HBP no-hypointensity	0.735 (0.139, 3.880)	0.717		
Peritumoral hypointensity on HBP	8.267 (2.926, 23.358)	< 0.001*	4.646 (1.303, 16.569)	0.018*
Targetoid TP or HBP appearance	–	–		
Size of the largest tumor(cm)	1.259 (1.101, 1.440)	0.001*		
Age (years)	0.967 (0.933, 1.003)	0.068		
ALT (IU/l)	1.103 (0.997, 1.110)	0.284		
AST (IU/l)	1.012 (1.000, 1.025)	0.049*	1.013 (1.000, 1.027)	0.056
PT(s)	1.010 (0.701, 1.456)	0.955		
PLT (× 10^9/l)	1.005 (0.999, 1.010)	0.086		
Gender (male/female)	0.850 (0.303, 2.386)	0.758		
AFP(> 400 ng/mL)	3.576 (1.568, 8.159)	0.002*	3.559 (1.294, 9.788)	0.013*
CEA (> 5 ng/mL)	3.160 (0.897, 11.130)	0.073		
HBsAg (Positive)	2.779 (0.971, 7.956)	0.057		
ALBI grade				
A	Reference			
B	1.410 (0.575, 3.456)	0.452		
Child-pugh				
A	Reference			
B	2.371 (0.144, 38.990)	0.546		

^*^referred to *p* < 0.05; *HBP* Hepatobiliary phase, *APHE* Arterial phase hyperenhancement, *PVP, T2WI* T2-weighted imaging, *TP* Transitional phase, *ALT* Alanine aminotransferase, *AST* Aspartate aminotransferase, *PT* Prothrombin time, *PLT* Platelet count, *AFP* Alpha-fetoprotein, *CEA* Carcinoma embryonic antigen, *HbsAg* Hepatitis B surface antigen, *ALBI* Albumin-bilirubin grade; Score = − 4.220 + 0.936*(Satellite lesions: Yes: 1; No: 0) + 0.875*(Corona enhancement: Yes: 1; No: 0) + 0.966* (Single nodule type with extra-nodular growth: Yes: 1; No: 0) + 1.536* (Peritumoral hypointense in HBP: Yes: 1; No: 0) + 1.270* (AFP (> 400 ng/mL): Yes: 1; No: 0) + 0.013*AST(IU/L); threshold value = − 0.835

### Predictive performance of preoperative models

**Fig. 3:**
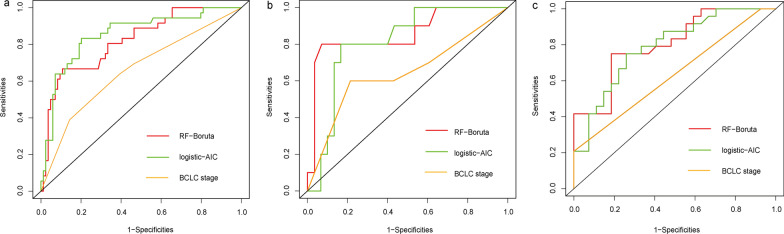
Receiver operating characteristic analysis for the prediction models. **a** Training Set; **b** Internal validation set; **c** External validation set. RF-Boruta, Random Forest model with Boruta algorithm; Logistic-AIC, Logistic regression model with stepwise selection (Akaike information criterion); BCLC, Barcelona Clinic Liver Cancer stage

Finally, two models have been developed including one random forest model with Boruta algorithm (RF-Boruta), and one logistic regression model with stepwise selection method(logistic-AIC). In the training cohort, the Logistic-AIC model provided the best discrimination performance in the training set (AUC, 0.853;95% CI, 0.775–0.930), which was followed by the RF-Boruta model (AUC, 0.820;95% CI, 0.738–0.902). However, no significant difference was obtained between these two models (*p* = 0.569). Compared with the BCLC stage, both the two models achieved significantly higher AUC values (*p* < 0.001). In the internal validation cohort, only the RF-Boruta model demonstrated a significantly better discrimination power than the BCLC stage (RF-Boruta: AUC, 0.857; 95% CI, 0.699–0.999 vs. BCLC stage: AUC, 0.643; 95% CI, 0.422–0.863, *p* = 0.038). While the RF-Boruta model and the logistic-AIC model showed significantly better discrimination power than the BCLC stage ((RF-Boruta: AUC, 0.805; 95% CI, 0.686–0.924 vs. logistic-AIC: AUC, 0.789; 95% CI, 0.665–0.913 vs. BCLC stage: AUC, 0.633; 95% CI, 0.547–0.720, *p* < 0.05). The diagnostic performance and ROC analysis of the two models and the BCLC stage were shown in Table 4 and Fig. 3, respectively.

**Table 4:** Prediction performance of the two developed models and the BCLC stage

Models	Training set (*n* = 120)			Internal validation set (*n* = 38)			External validation set (*n* = 51)		
	AUC (95% CI)	Sensitivity	Specificity	AUC (95% CI)	Sensitivity	Specificity	AUC (95% CI)	Sensitivity	Specificity
RF-Boruta	0.820 (0.738, 0.902)^*^	66.67% (24/36)	89.29% (75/84)	0.857 (0.699, 0.999)^**^	80.00% (8/10)	89.28% (25/28)	0.805 (0.686, 0.924)^^^	41.67% (10/24)	92.59% (25/27)
Logistic-AIC	0.853 (0.775, 0.930)^*^	83.33% (30/36)	79.76% (67/84)	0.812 (0.669, 0.955)	70.00% (7/10)	83.33% (23/28)	0.789 (0.665, 0.913)^^^	75.00% (18/24)	70.37% (19/27)
BCLC stage	0.658 (0.553, 0.762)	63.89% (23/36)	60.71% (51/84)	0.643 (0.422, 0.863)	60.00% (6/10)	57.14% (16/28)	0.633 (0.547, 0.720)	20.83% (5/24)	100.00% (27/27)

^*^:vs the BCLC stage in the training set, *p* < 0.001; ^**^: vs the BCLC stage in the internal validation set, *p* = 0.038; ^: vs the BCLC stage in the external validation set, *p* < 0.05; RF-Boruta, the Random Forests model with Boruta algorithm; Logistic-AIC, the Logistic regression model with stepwise selection (Akaike information criterion); BCLC, Barcelona Clinic Liver Cancer stage

The Hosmer–Lemeshow test yielded non-significant results for the two models and the BCLC stage in the training set (all *p* > 0.05), and it demonstrated that both models and the BCLC stage had good calibration power. Whereas the BCLC stage overestimated the recurrence risk of patients in the internal validation set and external validation set (*p* < 0.001) and showed poor calibration performance (Additional file 1: Fig. E4).

Compared with the reference model (the BCLC stage), the net benefit for the RF-Boruta model and the logistic-AIC model were greater over the range of threshold probabilities (training set: about in the range of 0.18–0.64; internal validation set, about in the range of 0.22–0.58; external validation set, about in the range of 0.40–0.98) (Additional file 1: Fig. E5).

### Prognostic value of preoperative models

**Fig. 4:**
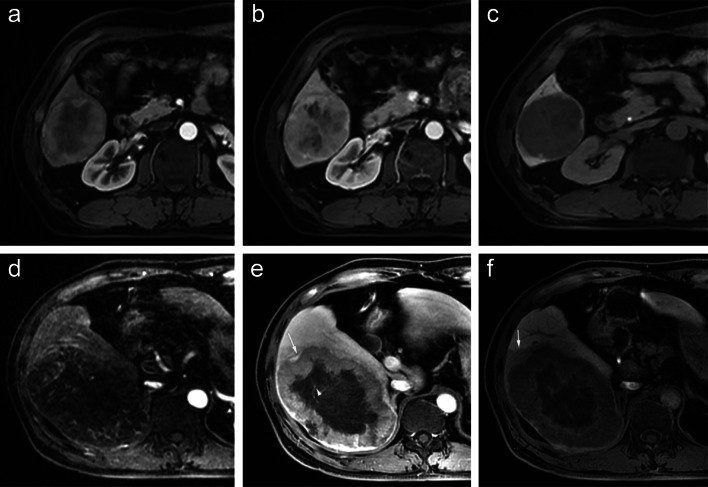
The representative patients with low risk and high risk of early postoperative recurrence. A 57-year-old woman with a 7.2 cm moderately differentiated HCC in hepatic segment V/VI, serum AFP level of 4.11 ng/mL, ALT level of 33 IU/l and AST level of 23 IU/l was divided into low risk group of Early postoperative recurrence after curative resection. The mass of this patient was a single nodule which showed non-rim hyperenhancement on arterial phase (**a**), no washout on portal venous phase (**b**) and hypointensity on hepatobiliary phase (**c**). This patient did not undergo recurrence during one-year follow up. A 44-year old man, who was identified with high risk of early postoperative recurrence, showed a 13.3 cm poorly differentiated HCC in hepatic segment VI/VII, serum AFP level of 706.5 ng/mL, ALT level of 40 IU/l and AST level of 64 IU/l. A mass of this patient showed non-rim hyperenhancement on arterial phase (**d**), non-rim washout, corona enhancement(arrow) and tumor in vein(arrowhead) on portal venous phase (**e**). Besides, it was a single nodule with extra-nodular growth, which showed hypointensity and peritumoral hypointensity (arrow) on hepatobiliary phase (**f**). The recurrence-free survival of this patient was 7.4 month

**Fig. 5:**
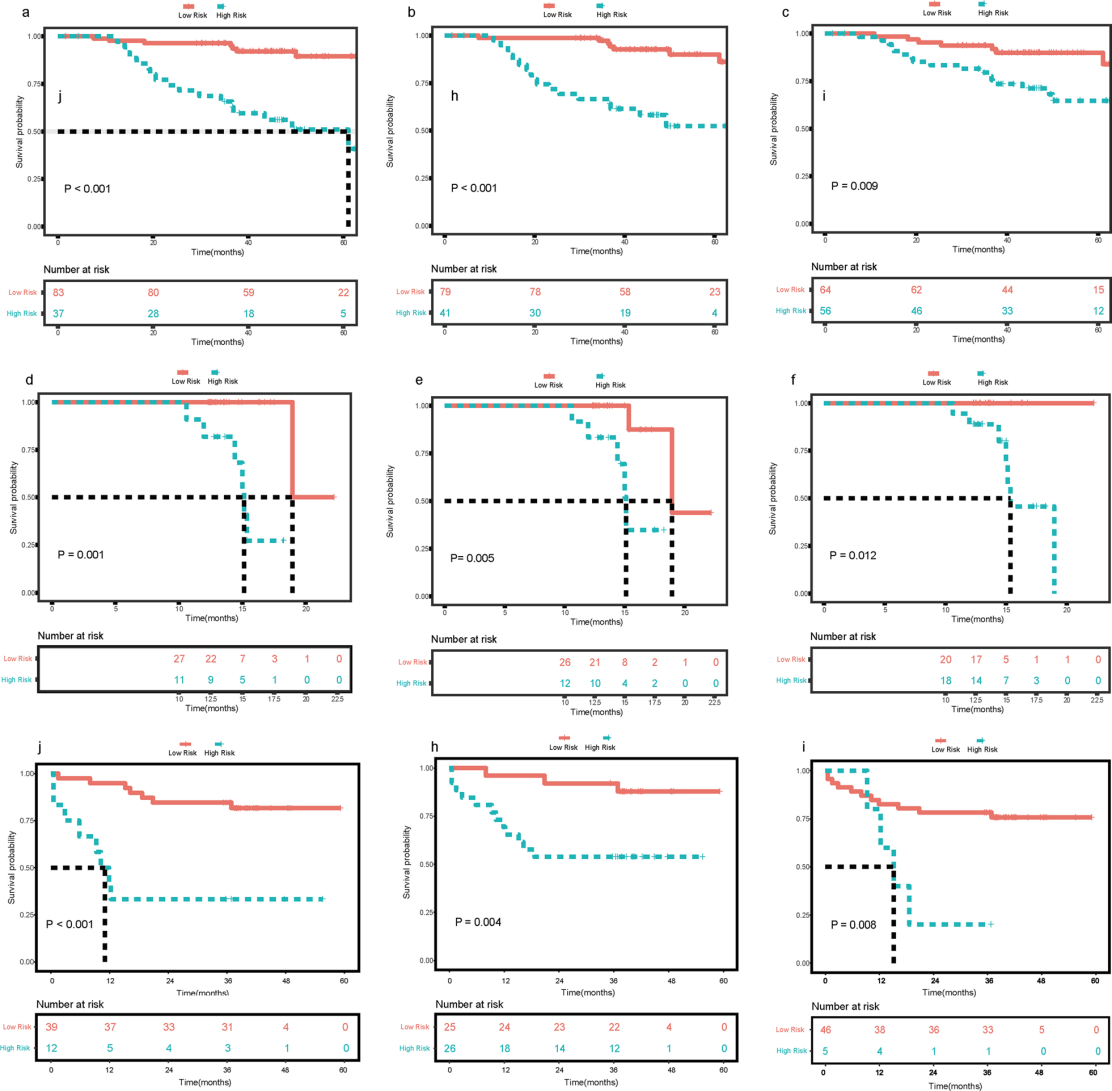
Kaplan–Meier curves of patients’ overall survival in Early postoperative recurrence and Non- Early postoperative recurrence groups classified by models. **a**–**c**: Training Set; **d**–**f**: Internal validation Set; **g**–**i**: external validation set. (**a**, **c**, **g**) the Random Forest model with Boruta algorithm; (**b**, **e**, **h**) the Logistic regression model with stepwise selection method (Akaike information criterion); (**c**, **f**, **i**) the BCLC stage

According to the risk of early postoperative recurrence classified by the two developed models (Fig. 4) and the BCLC stage, whether in the training or internal validation or external validation cohort, the group with a low risk of recurrence always had a longer survival duration than those with a high risk of early postoperative recurrence (all *p* < 0.05, Fig. 5).

## Discussion

In this study, the two preoperative prediction models, the random forest with Boruta algorithm and the logistic regression model with the stepwise selection method, have been developed using clinical and imaging features. Both the new models showed good discrimination performance. In particular, the RF-Boruta model demonstrated a significantly higher discrimination power than the BCLC stage whether in the training set, internal validation set or external validation set. Moreover, in accordance with the BCLC stage, a well-known tool for prognostic stratification in clinical practice, patients with low risk classified by the two new models lived longer than those with high risk. Hence, the preoperative prediction models may act as effective tools for prognostic stratification of early postoperative recurrence and OS for HCC patients.

Our results found that peritumoral hypointensity on HBP, satellite lesions, corona enhancement, single nodular type with extra-nodular growth, and AFP were important predictors of early postoperative recurrence in the RF-Boruta model and the Logistic-AIC model, even though the two models were constructed using a different method for variables selection. The potential reasons can be listed as follows: Firstly, the possible mechanism of peritumoral hypointensity on HBP is that peritumoral perfusion change, caused by the microvascular invasion, may result in a decreased uptake of gadoxetic acid in peritumoral hepatocytes [15]. A previous study [16] has proved that peritumoral hypointensity on HBP was an effective imaging biomarker of MVI, which was also a well-known risk factor of early postoperative recurrence in HCC. Besides, corona enhancement points to the draining of contrast material from hypervascular HCCs into the peritumoral hepatic parenchyma, and it was often accompanied by microvascular invasion and micro-metastasis occurrence [17]. The micro-metastasis usually manifested as satellite nodules deriving from the main tumor via the portal system, and the two imaging findings have been reported to be in good accordance with very early recurrence in HCC [18].

Considering Eggel’s growth classification, our study found that type 2 (the single nodular type with extra-nodular growth) was a risk factor for early recurrence. And it corresponded well with Hui’s study [19], because type 2 could reflect the emergence of cellular populations with higher proliferative and invasive activities, and it often has more malignant behavior. However, type 3 was not a risk factor for early postoperative recurrence in the final model, a possible explanation may be that our study population all underwent liver resection. In fact, many tumors in type 3 were unresectable HCC. Thus, the patients with type 3 tumors (Contiguous multinodular type) in our study may have relatively good prognostic outcomes, resulting in a nonsignificant outcome. Consistent with our study, a previous study [20] has reported that AFP level > 400 ng/mL was a risk factor for early recurrence of HCC, and the reason may be that elevated AFP level is associated with proliferation, growth acceleration and metastasis of HCC cells.

For the predictors only included in the variables set from Boruta algorithm, tumor size has been considered a marker for tumor invasiveness, which has proven to be a risk factor of higher histologic grade and the presence of MVI [21]. Accordingly, tumor size is associated with the early recurrence of HCC after resection [5]. However, the optimal tumor size for risk stratification remains somewhat controversial [22]. In this study, we did not classify tumor size into a categorical variable with a given cut-off value. An et al. [5] suggested that tumor size, as a continuous variable, was independently associated with early recurrence of HCC. Similarly, Chan et al. [23] has developed the EASL model to predict early recurrence of HCC after surgical resection and achieved better discrimination performance than the AJCC TNM stage, where tumor size was also shown as a continuous variable. ALT is a well-known biomarker of inflammatory necrosis in the liver. Persistent inflammatory necrosis will cause the regeneration of hepatocytes and gene instability, resulting in the frequent occurrence and intrahepatic metastasis of HCC [24]. Consistent with this study, a previous study [25] have reported elevated perioperative ALT level was an independent risk factor for the recurrence of HCC. However, a study [26] has found the old cut-off value (40 U/L) was questionable for that some patients with persistently normal levels displayed severe liver inflammation and fibrosis. Besides, the levels of ALT can be affected by age and gender [27]. Hence, we used the original value of ALT as a predictor in this study, rather than changing it into a categorical variable. Tumor in vein is the direct evidence of tumor invasiveness, which was associated with more aggressive tumor behavior and higher-grade HCCs [28], and it has been reported to be a critical risk factor for early postoperative recurrence of HCC [7].

In this study, AST was the predictor only included in the logistic regression model with stepwise selection. Apart from ALT, AST is also a reliable and sensitive biochemical indicator to reflect the environment of repeated chronic inflammation or the progression of hepatic fibrosis, leading to the enhancement of proliferating activity (mitosis) and the development of new foci of HCC [29]. Preoperative level of serum AST was statistically significant for predicting early recurrence of HCC in a previous study [30]. AST values for men is generally higher than for women up to age 65 [31], and it is still in dispute of the optimal cut-off value of AST. Consistent with our study, Muhi et al. [32] proved that AST, a continuous variable, was an independent risk factor for early postoperative recurrence after surgical resection for HCC. A possible explanation for the different variables selected by the two algorithms is that the stepwise selection method was based on the linear association between variables and outcomes, while Boruta algorithm, a random forest classification framework using bootstrap validation, enables to address complex nonlinear association and identify a minimal set of relevant variables strongly relevant to the outcome [13]. Evidence [33] has proved that Boruta algorithm could reduce the misleading impact of random fluctuations, avoid over-fitting, and improve model generalizability by correction. Accordingly, in this study, the RF model with Boruta algorithm had good generalization performance with the best AUC value in the test set.

The two new models (RF-Boruta and Logistic-AIC) obtained better performance than the BCLC stage in predicting early postoperative recurrence of HCC, and they could also work as effective tools to provide prognostic stratification of overall survival for HCC patients. Moreover, the RF-Boruta model without radiomics features in this study yields similar discrimination power (Internal validation set AUC: 0.854; 95% CI, 0.699–0.999; External validation set: 0.805; 95% CI, 0.686–0.924) with the radiomics nomogram in Zhang et al. [7] (AUC, 0.841; 95% CI, 0.722–0.959). Hence, this proposed RF-Boruta model could assist clinicians to optimize the treatment decision-making process according to the risk of early postoperative recurrence. Specifically, for patients with a high risk of early postoperative recurrence, they could choose the liver transplantation or adjuvant therapy rather than liver resection, while patients with a low risk of early postoperative recurrence might be the surgical candidates.

## Limitations

This study has several potential limitations. First, although the patients in our study were from two different institutions, the sample size of our study was relatively small, which may limit the generalization of the prediction models. Second, the follow-up time was relatively short: the follow-up time of some patients in the internal validation set was less than 2 years, and the data of these surviving patients were censored. Third, in our study, we only included patients undergoing initial hepatectomy. For recurrent patients undergoing repeated hepatectomy, it is also indispensable to assess its prognostic outcome and go on precise decision-making in clinical practice. Hence, in the future, a prospective study with large cohorts from different institutions and a longer follow-up period is warranted to evaluate the role of MR imaging features in predicting early recurrence of HCC and long-term survival. In addition, we could also include patients undergoing repeated hepatectomy and develop a prediction model of early postoperative recurrence in recurrent HCC patients.

## Conclusion

In conclusion, two predictive models for early postoperative recurrence prediction were developed based on preoperative clinical and MR findings. The two models, especially the RF-Boruta model, demonstrated good discriminatory performance and generalization ability in early postoperative recurrence prediction and prognostic stratification. Therefore, they could be effective tools to assist clinicians in considering more optimal and comprehensive treatment approaches, and could further improve the overall survival of patients with HCC after resection.

## Supplementary Information

**Additional file 1**. **ESM 1**: MRI Sequences and Parameters. **ESM 2**: The definitions of the evaluated imaging findings. **ESM 3**. **ESM 4**.

## Data Availability

The data are available for scrutiny from external requests. Competing interests: All of the authors declare that there are no conflicts of interest.

## Abbreviations

AIC: Akaike information criterion
AFP: Alpha-fetoprotein
AUC: Area under the receiver operating characteristic curve
CEA: Carcinoembryonic antigen
DCA: Decision curve analysis
GBDT: Gradient-boosted decision tree
HBP: Hepatobiliary phase
HCC: Hepatocellular carcinoma
IQRs: Interquartile ranges
logistic-AIC: Logistic regression model with stepwise selection method based on Akaike information criterion
MR: Magnetic resonance
OS: Overall survival
RF: Random forest
RF-Boruta: Random forest model with Boruta algorithm
ROC: Receiver operator characteristic
