# Supplementary material for: Predictive models and early postoperative recurrence evaluation for hepatocellular carcinoma based on gadoxetic acid-enhanced MR imaging
Source: Insights Imaging. 2023 Jan 8;14:4. doi: 10.1186/s13244-022-01359-5 (PMC9826770; doi:10.1186/s13244-022-01359-5)
Supplement: Supplementary file 1 — Additional file 1. ESM 1: MRI Sequences and Parameters. ESM 2: The definitions of the evaluated imaging findings. ESM 3. ESM 4. [file 13244_2022_1359_MOESM1_ESM.pdf]

ELECTRONIC SUPPLEMENTARY MATERIAL

Predictive models and early postoperative recurrence evaluation for Hepatocellular Carcinoma Based on gadoxetic acid-enhanced MR imaging

ESM 1: MRI Sequences and Parameters

Sequence	Fat suppression	TR (ms)	TE (ms)	Flip angle	Section thickness (mm)	Matrix size	Field of view (mm^2)	Acquisition time (s)
T2-weighted 2D TSE	Used	2160	100	160°	6	320×288	433×433	36
Coronal T2-weighted HASTE	Used	1000	96	129°	3	320×320	400×400	25
T1-weighted 3D GRE VIBE	Used	3.95	1.92	9°	2	352×256	400×296	14
T1-weighted IP and OP imaging	None	81	1.4	70°	6	352×286	400×325	24
DW single-shot spin-echo EPI	Used	5600	68	90°	6	100×76	380×289	233

DW diffusion-weighted, EPI echo planar imaging, GRE gradient recall echo, HASTE half fuorier single-shot turk spine-echo, IP in-phase, MRI magnetic resonance imaging, OP opposed-phase, TE echo time, TR repetition time, TSE turbo spin-echo, VIBE volume interpolated breath-hold examination, 2D two-dimensional, 3D three-dimensional

ESM 2

MR imaging feature	Definitions
**Tumor size**	The maximum diameter measured on PVP[1]
**Multifocality**	More than one nodule on PVP [1], and the additional nodule should not be satellite lesion.
**Arterial phase hyperenhancement**	
**no**	The tumor nodule in arterial phase is not enhanced
**rim**	Enhancement in arterial phase is most pronounced in observation periphery
**non-rim**	Non rim-like enhancement in arterial phase unequivocally greater in whole or in part than liver
**"Capsule" appearance**	
**no**	No capsule
**enhancing**	Smooth, uniform, sharp border around most or all of an observation, unequivocally thicker or more conspicuous than fibrotic tissue around background nodules, and visible as enhancing rim on PVP, DP, or TP
**non-enhancing**	Capsule appearance not visible as an enhancing rim
**Washout**	
**no**	No washout
**rim**	Apparent washout is most pronounced in observation periphery
**non-rim**	Non-peripheral visually assessed temporal reduction in enhancement in whole or in part relative to composite liver tissue from earlier to later phase resulting in hypoenhancement in the portal venous phase

**Nodule-in-nodule architecture**	Presence of smaller inner nodule within and having different imaging features than larger outer nodule
**Mosaic architecture**	Presence of randomly distributed internal nodules or compartments, usually with different imaging features
**Blood products in mass**	Intralesional or perilesional hemorrhage in the absence of biopsy, trauma or intervention
**Transitional phase hypointensity**	Intensity in the transitional phase unequivocally less, in whole or in part, than liver
**Restricted diffusion**	Intensity on DWI, not attributable solely to T2 shine -through, unequivocally higher than liver and/or ADC unequivocally lower than liver
**Mild-moderate T2 hyperintensity**	Intensity on T2WI mildly or moderately higher than liver and similar to or less than non-iron-overloaded spleen
**Corona enhancement**	Peri-observational enhancement in late arterial phase or early PVP attributable to venous drainage from tumor
**HBP intensity**	
**hypointensity**	Intensity in the hepatobiliary phase unequivocally less, in whole or in part, than liver
**isointensity**	Intensity in hepatobiliary phase nearly identical to liver
**hyperintensity**	Intensity in hepatobiliary phase unequivocally more, in whole or in part, than liver than liver
**Tumor in vein**	Unequivocal enhancing soft tissue in vein, regardless of visualization of parenchymal mass
**Non-smooth tumor margin**	irregular margin that had budding portion at the tumor periphery on HBP[2]
**Peritumoral hypointensity on HBP**	A wedge-shaped or flamelike hypointense area of hepatic parenchyma surrounding the tumor on HBP[3]

**Presence of non-hypervascular HBP hypointense nodules**	Solid nodules greater than 3.5 mm in diameter with low signal intensity on HBP, which do not show higher signal intensity than that of the spleen on heavily T2-weighted images[4]
**Satellite lesions**	tumors≦2 cm in size and located ≦2 cm from the main tumor[5]
**Incomplete capsule**	A capsule only partly surrounding the tumor border[6]
**Eggel’s growth classification**	
**single nodular**	A roughly round tumor with a clear demarcation
**single nodular with extranodular growth**	A tumor-like single nodular type but showing extranodular growth
**contiguous multinodular**	A tumor formed by a cluster of small and contiguous nodules
**Targetoid TP or HBP appearance**	Concentric pattern in TP or HBP characterized by moderate-to-marked hypointensity in observation periphery with milder hypointensity in center
**Cirrhosis**	Surface nodularity, generalized widening of the interlobar fissures, an expanded gallbladder fossa, the notch-sign of the right lobe, and segmental hypertrophy involving the lateral segments of the left lobe and caudate lobe[7]

HCC was considered single when nodules close to the primary tumor were designated as satellite nodules; otherwise, ICCs were considered multiple. For patients with multiple tumors, all measurable observations were assessed and the largest observation was selected as the representative for statistical analysis. Except for the extra indications, all definitions of MR imaging features refer to the LI-RADS v2018 guideline [8]

MR: magnetic resonance; PVP: portal venous phase; DP: delayed phase; TP: transitional phase; DWI: diffusion-weighted imaging; ADC: apparent diffusion coefficient; T2WI: T2-weighted imaging; HBP hepatobiliary phase, LI-RADS Liver Imaging Reporting and Data System.

1. Zhang Z, Jiang H, Chen J, et al. (2019) Hepatocellular carcinoma: radiomics nomogram on gadoxetic acid-enhanced MR imaging for early postoperative recurrence prediction. Cancer imaging : the official publication of the International Cancer Imaging Society 19:22-22.
2. Ariizumi S, Kitagawa K, Kotera Y, et al. (2011) A non-smooth tumor margin in the hepatobiliary phase of gadoxetic acid disodium (Gd-EOB-DTPA)-enhanced magnetic resonance imaging predicts microscopic portal vein invasion, intrahepatic metastasis, and early recurrence after hepatectomy in patients with hepatocellular carcinoma. J Hepatobiliary Pancreat Sci 18:575-585.
3. Kim KA, Kim MJ, Jeon HM, et al. (2012) Prediction of microvascular invasion of hepatocellular carcinoma: usefulness of peritumoral hypointensity seen on gadoxetate disodium-enhanced hepatobiliary phase images. J Magn Reson Imaging 35:629-634.
4. Toyoda H, Kumada T, Tada T, et al. (2013) Non-hypervascular hypointense nodules detected by Gd-EOB-DTPA-enhanced MRI are a risk factor for recurrence of HCC after hepatectomy. J Hepatol 58:1174-1180.
5. Roayaie S, Blume IN, Thung SN, et al. (2009) A system of classifying microvascular invasion to predict outcome after resection in patients with hepatocellular carcinoma. Gastroenterology 137:850-855.
6. Kim H, Park MS, Choi JY, et al. (2009) Can microvessel invasion of hepatocellular carcinoma be predicted by pre-operative MRI? Eur Radiol 19:1744-1751.
7. Brancatelli G, Federle MP, Ambrosini R, et al. (2007) Cirrhosis: CT and MR imaging evaluation. Eur J Radiol 61:57-69.
8. Inoue Y, Fujii K, Ishii M, et al. (2019) Volumetric and Functional Regeneration of Remnant Liver after Hepatectomy. J Gastrointest Surg 23:914-921.

ESM 3

Figure E1. Kaplan-Meier curves of overall survival of patients with or without early postoperative recurrence in the Training set(a),internal validation set(b) and external validation set(c).

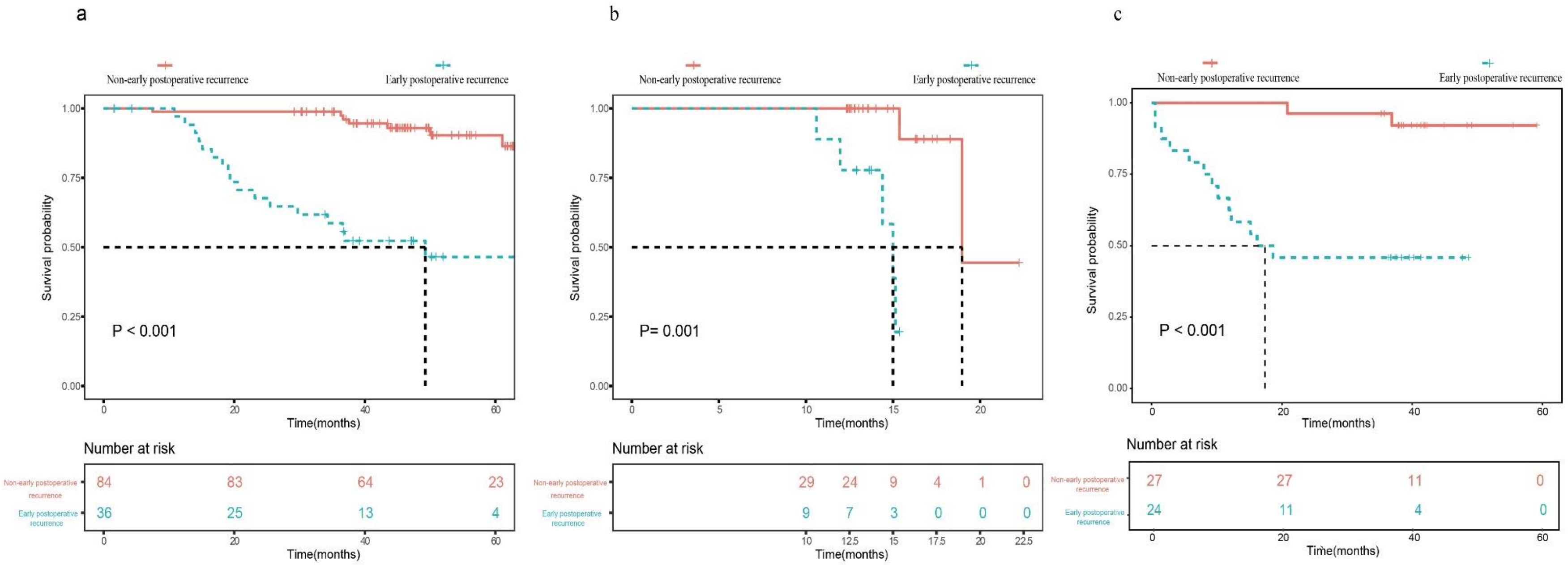

Figure E2. Kappa values and Kendall’s value for inter-observer agreement assessment

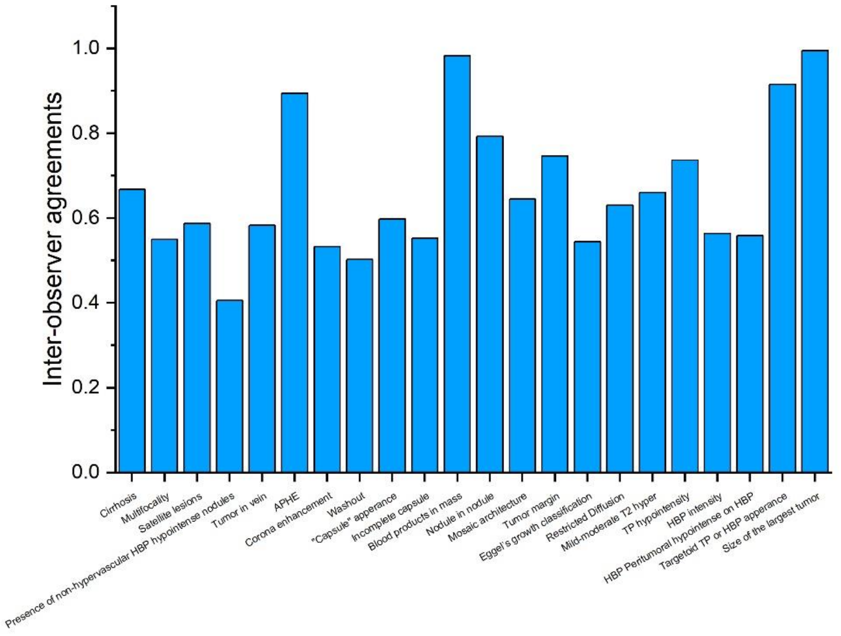

HBP, Hepatobiliary phase; APHE, Arterial phase hyperenhancement; TP, Transitional phase

Figure E3. Feature selection based on Boruta Algorithm

a b

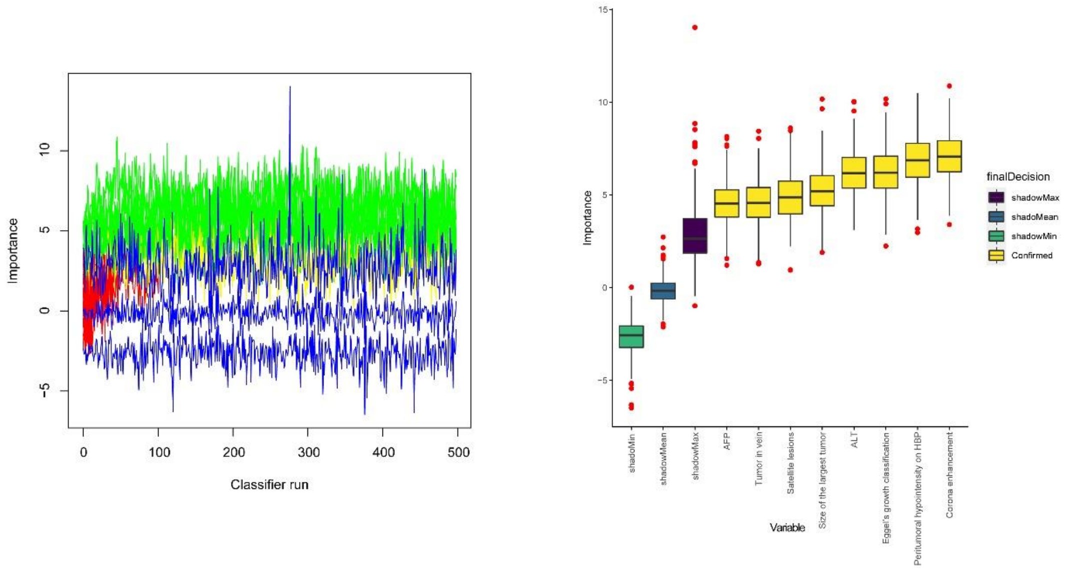

a: the number for classifier run. The numbers under the x-axis indicate the times of classifier run, the y-axis is the importance of predictors, vertical lines represent three types of features (blue: shadow features (max, mean, min); green, confirmed features; yellow, tentative features). When classifier run 500 times, the importance of confirmed feature is obviously higher than those of shadow features and tentative features. b: Feature selection based on Boruta algorithm, the y-axis denotes the importance of feature. The green boxplot, blue boxplot and purple boxplot correspond to minimal, average, and maximum Z score distribution of the shadow features, respectively. Shadow features mean the shuffled copies of all original features mixed with randomly added values. The yellow boxplots represent Z scores of confirmed features after deleting reject and tentative features. The importance of confirmed features is higher than the maximal importance shadow feature.

Figure E4. Calibration plots of prediction models

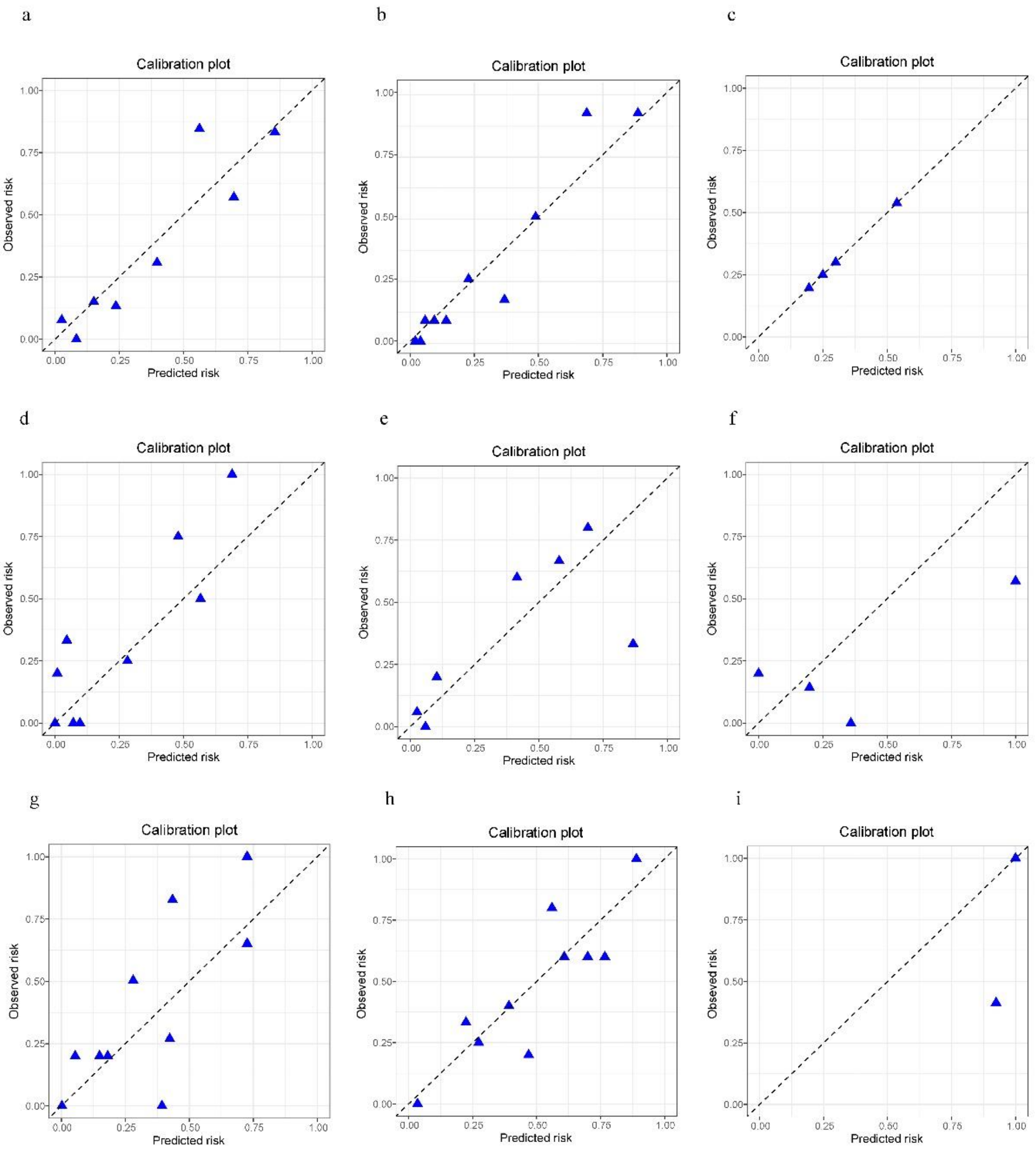

a-c: Training Set; d-f: Internal validation Set; g-i: External validation set; (a, d, g) the Random Forest model with Boruta algorithm; (b, e, h) the logistic regression model with stepwise selection method (Akaike information criterion); (c, f, i) the Barcelona Clinic Liver Cancer stage.

Figure E5. Decision curve analysis of Prediction models

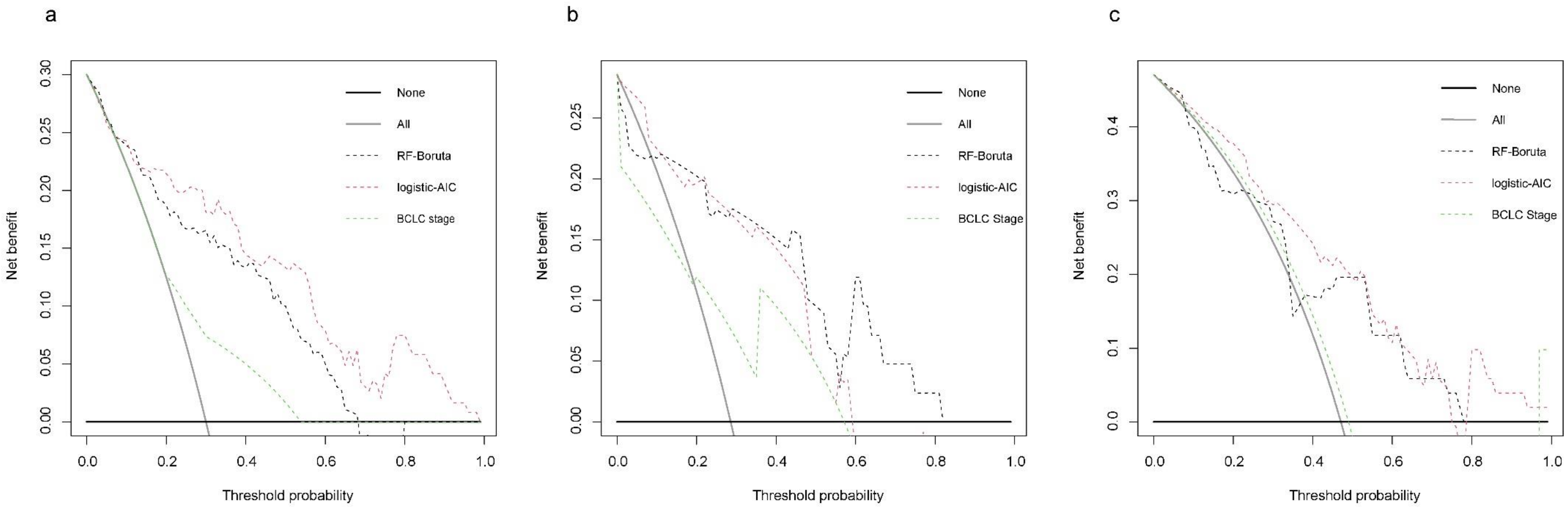

(a) Training Set; (b) Internal validation Set; (c) External validation Set; RF-Boruta: the Random Forest model with Boruta algorithm; Logistic-AIC, the logistic regression model with stepwise selection method (Akaike information criterion); BCLC, Barcelona Clinic Liver Cancer stage. The x-axis indicates the threshold probability. The y-axis indicates the net benefit. The decision curves indicate the net benefit of models as well as 2 clinical alternatives (classifying no patients as undergoing early postoperative recurrence vs classifying all the patients as undergoing ER) over a specified range of threshold probabilities of early postoperative recurrence. Compared with the reference model (the BCLC stage), the net benefit for the Random Forest, and Gradient-Boosted Decision Tree model were greater over the range of threshold probabilities (training Set: about in the range of 0.18-0.64; Internal validation Set, about in the range of 0.22-0.58; External validation Set, about in the range of 0.40-0.98)

ESM 4

The Best Combination of Hyperparameter of Machine learning Models and Models selection

The best combination of Hyperparameter of Machine learning Models

Random Forest (RF)

*With 10-fold cross-validation repeated five times in the training group, the maximum AUC of RF model appeared when using 200 trees and 1 predictor. (**Figure S1**).*

Gradient-Boosted Decision Tree (GBDT)

*The GBDT model achieved the maximum AUC value with the same combination of hyperparameter (maximum depth=1, number of iterations=50, eta=0.3, subsample=0.6667 and colsample_bytree=0.6) (**FigureS2**).*

Selection of Prediction Models using 10-fold Cross-validation

*Ultimately, based on the variables set from Boruta algorithm, we have constructed 3 models (two deep learning models and one logistic regression model). And internal validation with 10-fold cross-validation in the training set revealed that the RF model achieved the better discrimination performance (AUC, 0.828; 95% CI, 0.743-0.944) when compared with the other two models (**TableS1**); hence, we trained RF model using variables set from Boruta algorithm to construct the final model (RF-Boruta) in the entire training group.*

The Variables importance in the Random Forest models

***Figure S3** demonstrates the variable importance in the RF model, the size of the largest tumor and peritumoral hypointense on HBP were the top two important predictors, And Eggel’s growth classification (contiguous multinodular type) was the least important predictor for early postoperative recurrence.*

Table S1. Prediction performance of the developed models in internal validation with 10-fold Cross-validation

Models	*AUC (95% CI)*	*Sensitivity (95% CI)*	*Specificity (95% CI)*
RF-Boruta	*0.828 (0.743,0.944)*	*55.50% (45.00%,70.00%)*	*85.11% (78.89%,90.00%)*
Logistics-Boruta	*0.816 (0.710,0.943)*	*65.00% (50.00%,75.00%)*	*77.14% (67.78%,90.00%)*
GBDT-Boruta	*0.799(0.711,0.943)*	*52.50% (33.33%,75.00%)*	*86.39% (75.69%,94.27%)*

RF-Boruta: the Random Forest model with Boruta algorithm; Logistic-Boruta, the Logistic regression model with Boruta algorithm; GBDT-Boruta, the Gradient-Boosted Decision Tree model with Boruta algorithm.

Figure S1. The number of predictors and the number of trees selection for the Random Forest model using 10-fold cross-validation repeated 5

a

b

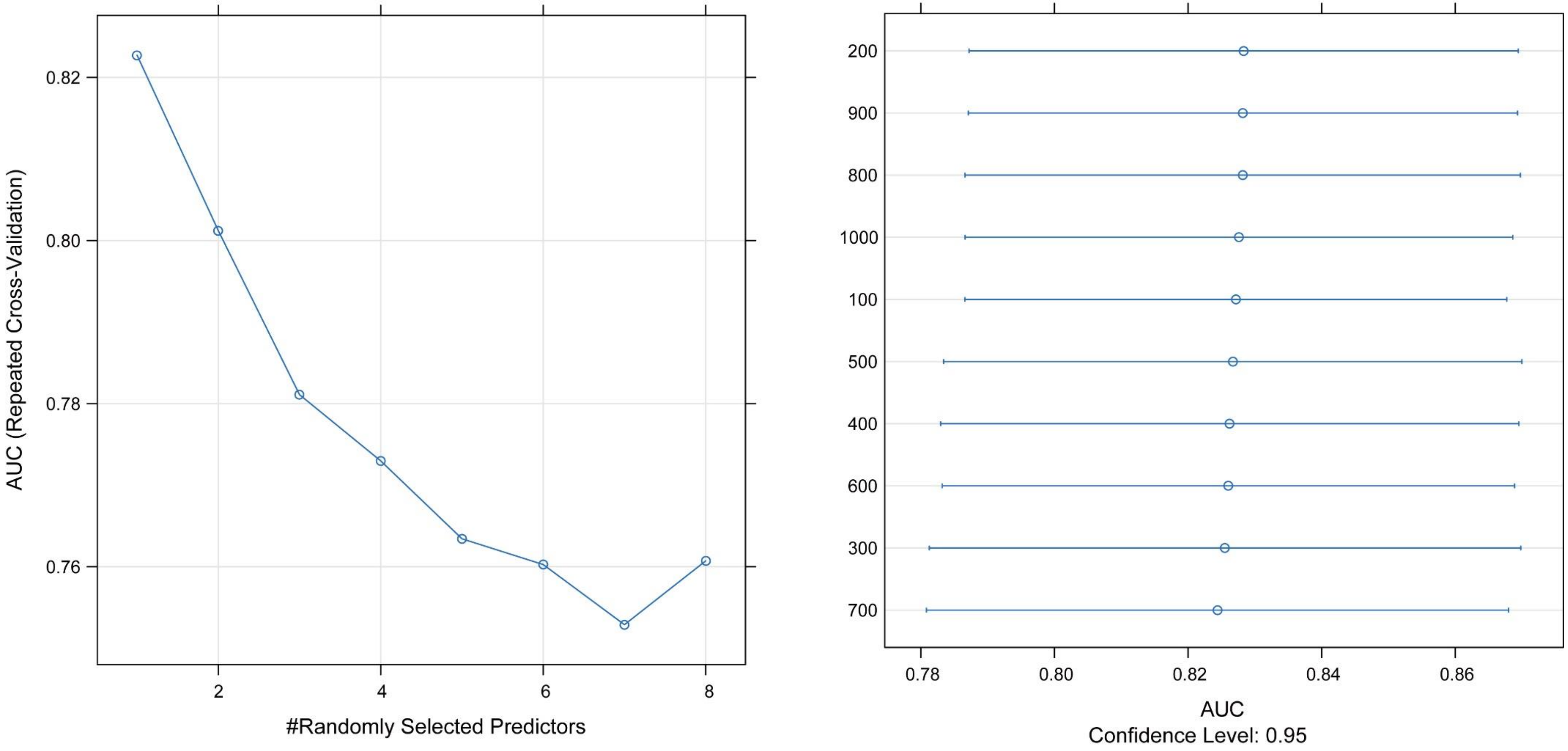

times

a, b: Optimal values of mry and ntree for the RF model using variable set from Boruta algorithm; A: The numbers along the lower x-axis indicate the number of predictors, The y-axis is the value of AUC, the broken line reveals the variation tendency of AUC value to the number of predictors using 10-fold cross-validation repeated 5 times, where the optimal AUC value resulted in one predictior; B: The numbers along the y-axis indicate the number of trees, the x-axis is the value of AUC, the horizontal line is drawn at the mean values of AUC and its 95% confidence interval at the relevant number of trees, where the optimal values of ntree with best AUC were 200.

Figure S2. The hyperparameter selection for the GBDT model using 10-fold cross-validation repeated 5 times

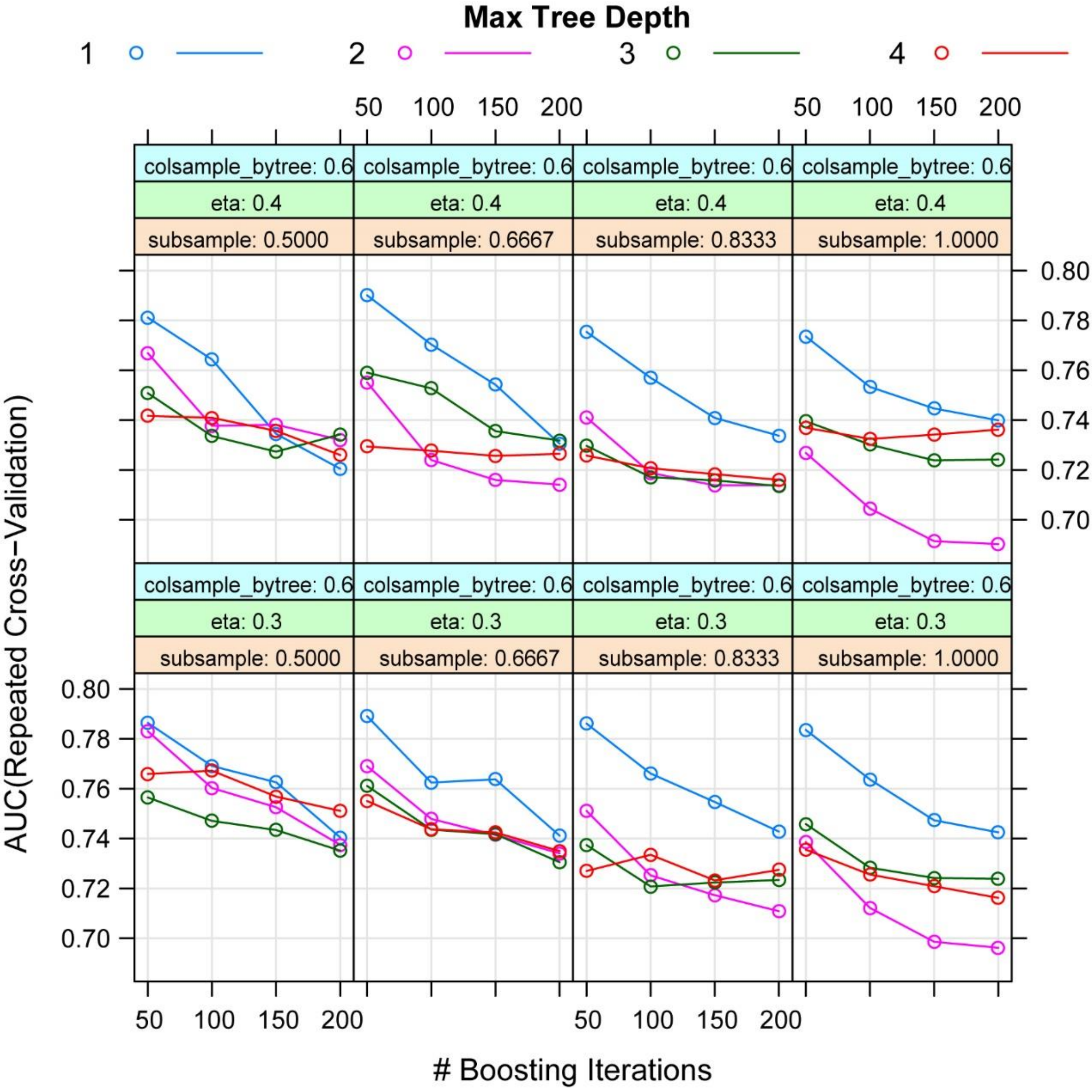

The Value of AUC was plotted vs. the number of iterations. The numbers along the upper x-axis indicate the number of iterations, The y-axis is the value of AUC, in the given value of colsample, bytree, eta and subsample, the broken line reveal the variation tendency of AUC value to the number of rounds using 10-fold cross-validation repeated 5 times, the maximum depth is in the range of one to four (1: blue line; 2: purple line; 3: green line; 4 :red line), where optimal AUC value resulted in the following selections (maximum depth=1, colsample, bytree=0.6, eta=0.3, subsample=0.6667, number of iterations=50).

Figure S3. Importance of Predictors From the Random Forest model for early postoperative recurrence

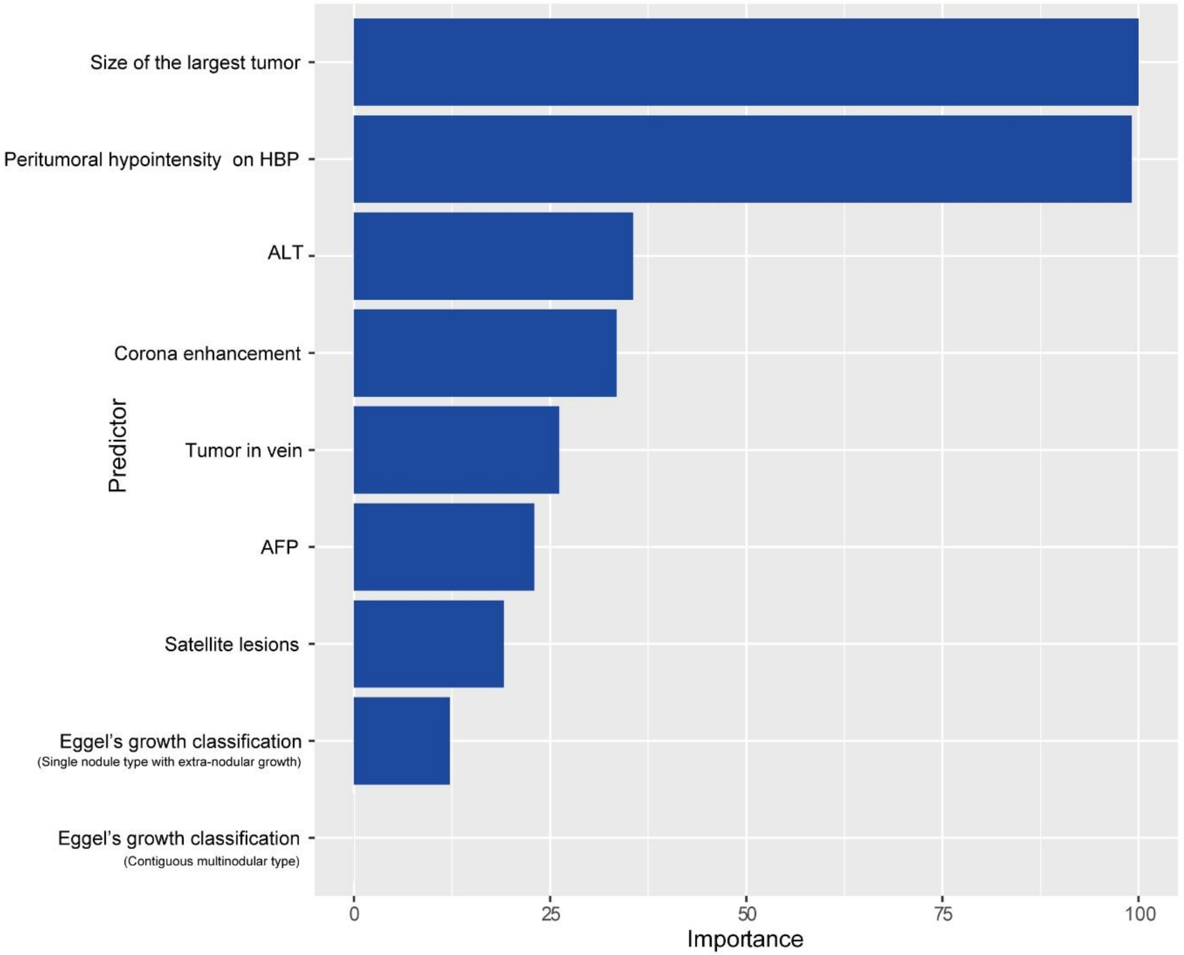

Relative influence values range from 0 to 100(y-axis) and indicate the proportional contribution of each variable(x-axis) in predicting early

postoperative recurrence of patients with HCC. The relative influence of the 8 final predictors for early postoperative recurrence were plotted from the Random Forest model.
